# The Relevance of Goodness‐of‐fit, Robustness and Prediction Validation Categories of OECD‐QSAR Principles with Respect to Sample Size and Model Type

**DOI:** 10.1002/minf.202200072

**Published:** 2022-07-25

**Authors:** Péter Király, Ramóna Kiss, Dániel Kovács, Amine Ballaj, Gergely Tóth

**Affiliations:** ^1^ Institute of Chemistry Loránd Eötvös University Pázmány S.1/A 1117 Budapest Hungary

**Keywords:** regression, sample size, modelling, external validation, internal validation

## Abstract

We investigated the relevance of the validation principles on the Quantitative Structure Activity Relationship models issued by Organization for Economic and Co‐operation and Development. We checked the goodness‐of‐fit, robustness and predictivity categories in linear and nonlinear models using benchmark datasets. Most of our conclusions are drawn using the sample size dependence of the different validation parameters. We found that the goodness‐of‐fit parameters misleadingly overestimate the models on small samples. In the case of neural network and support vector models, the feasibility of the goodness‐of‐fit parameters often might be questioned. We propose to use the simplest y‐scrambling method to estimate chance correlation. We found that the leave‐one‐out and leave‐many‐out cross‐validation parameters can be rescaled to each other in all models and the computationally feasible method should be chosen depending on the model type. We assessed the interdependence of the validation parameters by calculating their rank correlations. Goodness of fit and robustness correlate quite well over a sample size for linear models and one of the approaches might be redundant. In the rank correlation between internal and external validation parameters, we found that the assignment of good and bad modellable data to the training or the test causes negative correlations.

## Graphical abstract

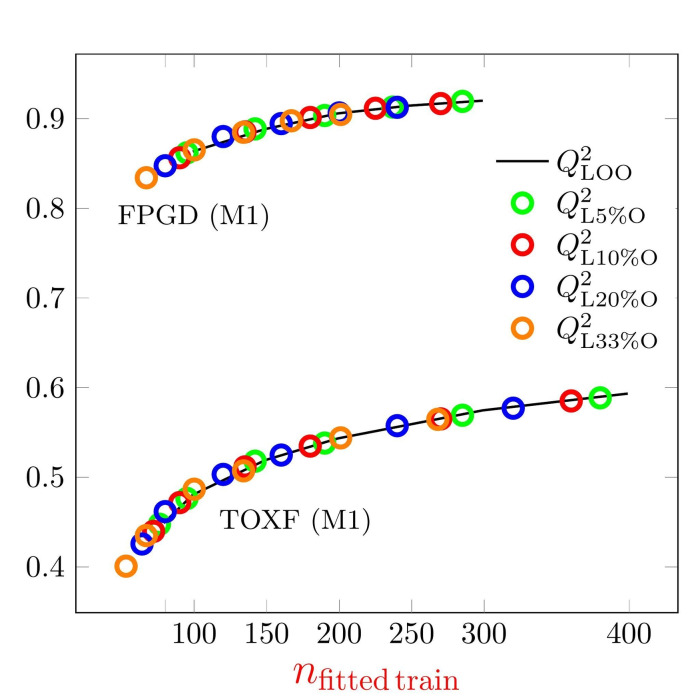

## 1 Introduction

Modelling in science seems to be essential to understand, predict and prefigure nature and processes. Since science should provide relevant and reproducible findings, it is necessary to validate models. The quantitative measures of this process are called validation parameters. Validation is usually performed using some basic principles (sometimes called standards), however, the concrete steps of a validation show great differences due to the general use of model building. Furthermore, there are also differences in the denomination or nomenclature of the processes, sometimes there are misleading differences even within a single field of application. At a workshop on QSAR (Quantitative Structure Activity Relationship) held in Setubal in 2002, the first international attempt at clarifying nomenclature and basing validation on a theoretically and methodologically sound foundation was made. These Setubal principles were the basis for a project which provided a regularization issued as OECD (Organization for Economic and Co‐operation and Development) principles^[1]^ in 2004 and a “Guidance document on the validation of (Quantitative) Structure‐Activity relationships[(Q)SAR] models” in 2007^[1]^ . It is discussed in some publications[^2^, ^3^, ^4^] and in a recent comprehensive study of Gramatica.^[5]^ The latter is an excellent overview of good practices concerning all the 5 OECD principles: 1) a defined endpoint 2) an unambiguous algorithm 3) a defined domain of applicability 4) appropriate measures of goodness‐of–fit, robustness and predictivity 5) a mechanistic interpretation, if possible. The 4^th^ OECD principle and the corresponding parts in the guidance clearly define the terms internal and external validations with providing the corresponding aims. In internal validation, the goodness of fit and robustness are assessed while external validation evaluates the predictivity of models.

A model might contain hyperparameters and parameters.[^6^, ^7^] We use the term hyperparameter for the different settings used to select the mathematical or operational form of the model. Sometimes, they are called meta‐ or tuning parameters, as well. For example, the number of latent variables or the optional standardization of data in biased linear regression methods are hyperparameters. In the case of, e.g., artificial neural networks (ANN), the structure of the network, the number of neurons in the hidden layers, the activity function, the optimization algorithm and their internal settings (tolerance limit, initial learning rates, etc…) are hyperparameters. On contrary, the simple ‘parameter’ term denotes the weights, slopes and intercept optimized in a direct or iterative calculation according to a well‐defined objective function and operation algorithm.

The OECD guidance classifies the data used in the parameter optimization as internal ones. Usually, we use the training set denomination for them. If not the entirety of the available data is used for the parameter optimization, there is a possibility to form an external test set to be used in external validation. According to the guidance, the internal set is used for two validation purposes: assessing goodness of fit and robustness. The validation parameters of goodness of fit are related to how well the model reproduces the response variables on which the parameters were optimized. The robustness is usually calculated by cross‐validation or bootstrap methods, where subsets or resampled sets of the training set data are used. In the case of cross validation, a reduced training set is used with smaller number of fitted data, *n*_fitted_, than the number of data in the training set, *n*_train_. In the case of bootstrapping, *n*_fitted_ and *n*_train_ are usually equal but resampling with repetition is allowed. In OECD terms, an external test set is defined as a set of data that are not used during the optimization of model parameters. Rather, an external test set is used to quantify predictivity. External test set based model selection from a pool of models with different hyperparameters is also a possibility.

However, the OECD 4th principle and the guidance do not state the weight of these three aspects in the overall quantification of model performance and how to select from the set of models having different hyperparameters. Of course, one might find recipes in the literature and their criticism as well.[^2^, ^5^, ^7^, ^8^, ^9^, ^10^, ^11^, ^12^, ^13^, ^14^, ^15^, ^16^, ^17^, ^18^]

In our antecedent study^[19]^ we showed how the relation among goodness‐of‐fit, robustness and predictivity validation parameters varied with respect to the sample size of the training set and the features of the datasets in the case of ordinary multivariate linear least‐squares regression (MLR). We showed on several datasets, that the goodness‐of‐fit parameters overrate models on small samples. We found that if there are no repetitions in the data, leave‐one‐out cross‐validation parameters and leave‐many‐out ones coincide on graphs, if the data are shown with respect to the fitted number of data during the cross‐validation procedure. We found that *x*‐ any *y*‐randomization methods are equivalent in the estimation of chance correlation. Using rank correlation, we found that internal and external validation parameters provide rather independent information from each other, but in the case of internal validation, goodness‐of‐fit and robustness measures highly correlate above an intermediate sample size for most of our datasets. Our results also question some elements in the large variability of methods and parameters in validation.

Our previous study raised the question whether the results are valid for other modelling methods, as well. In our actual study we show the extension of the MLR case to partial least squares regression with multiple responses (PLS2), to simple artificial neural network models (ANN) and to support vector machine regression (SVR). We focus on the common and the different features of the methods with respect to the OECD 4^th^ principle. The extension of our previous work to different model types can be summarized as checking the 4^th^ principle with respect to the 2^nd^ principle, where the question is the model type. In our study, we do not discuss the 1^st^ principle (endpoint), the 3^rd^ principle (domain of applicability) and the 5^th^ principle (interpretation). We think that the 5^th^ principle will be in the forefront during the next years to interpret the advanced models (ANN, SVR…) and to fill model parts and features with chemical and biological content.[^20^, ^21^]

Our investigation might be formulated also using the bias‐variance tradeoff. The tradeoff can be investigated pairwise concerning the model types. If linear relations are satisfactory for describing a model, e.g., MLR provides large variance for correlated variables, while PLS introduces a reasonable bias and reduces significantly the variance. If the correct number of latent variables is used, the error of predictivity decreases. In the case of ANN and SVR we will see, that both methods might provide close to perfect reproduction of training data. This implies high variance and overfitting in general, but the final performance of the models is rather good, because these methods are often able to correctly generalize the features of the training set. The bias‐variance tradeoff can be linked also to the sample size dependence, where data derived on small sample sizes are overfitted ones with large variance. This lack of generalisation decreases using large training set sizes. We note, that the bias‐variance tradeoff is an analogue of the precision/accuracy concept in analytical chemistry, but in analytical chemistry it is often possible to independently reduce both bias and variance.

As we mentioned, there is a high variability in the protocols as well as in the validation parameters. Most studies concerning validation parameters compare large numbers of different parameters (e.g., *Q* ^2^_F1_, *Q* ^2^_F2_, *Q* ^2^_F3_,[^22^, ^23^, ^24^] *F*‐types,^[25]^ Roy‐Ojha types,^[26]^ *CCC*,[^27^, ^28^, ^29^] etc… Both in our previous study and here, we faced this large amount of validation parameters. Finally, we decided to use here only two groups of them. The first group represents intensive parameters, we chose the coefficient of determination family, *R* ^2^‐*Q* ^2^_LOO_‐*Q* ^2^_LMO_‐*Q* ^2^_F2_. The second group contain extensive parameters, we show the results on the root mean square deviation (*RMSE*) like ones. We know that this restricted selection might bother several experts, but we think that the main aspects of the validity following the OECD QSAR validation guidance with respect to the modelling method and sample size can be understood in this way. We think that our investigation would be confusingly detailed, if further groups and variants of validation parameters would have been included here. If someone is interested in a wide bibliography and detailed discussion of the validation parameters, we refer to the reference lists of comprehensive studies.[^19^, ^30^]

In our previous study with MLR one aim was to check the performance of several validation parameters. In the case of the goodness of fit, there are no large differences between QSAR and other scientific applications, the most popular ones are *R* ^2^ (without adjustment) and *RMSE*. In the case of robustness in QSAR, mostly cross‐validation is used in leave‐one‐out (LOO), leave‐many‐out (LMO) and k‐fold ways. Double cross validation can be used also in QSAR (discussed, e.g., by Baumann and Baumann^[31]^) In this study we used only simple LOO and LMO. On the contrary, there is a large variety of predictivity parameters. For example, Roy et al. proposed to use mean absolute error (*MAE*) and showed an enhanced stability of *Q* ^2^_F2_ values calculated on data with omission of given percentiles from the tails.^[32]^ Chirico and Gramatica^[29]^ proposed to use *CCC* (correlation concordance coefficient) of Lin.[^27^, ^28^] In this study we selected *Q* ^2^_F2_ and *RMSE*_test_ to remain within the *R* ^2^‐*RMSE* families for predictivity, as well. We did not switch to *MAE* to avoid mismatch of root mean square and absolute value like errors. We investigated *CCC* in our previous paper,^[19]^ and we found that the advantages reported in the original papers of Lin were based on a confusion between *R* ^2^ and the square of Pearson's correlation coefficient (see supplementary information in [19]). It reduces our confidence despite the suggestions of [29]. We were assured in our MLR study, that *Q* ^2^_F3_ is a more stable measure than *Q* ^2^_F2_. It prevents the use of tricks during the test set allocation and provides a stability for small test/train ratios. In spite of these, we finally decided to use *Q* ^2^_F2_: a) It is easily interpretable, because the ratio contains sums of squares. For example, a zero value implies that the model has as much relevance as the use of mean values. b) It is generally used outside the QSAR field known as *R* ^2^_test_, or *R* ^2^_pred_. c) For *Q* ^2^_F3_ we proposed a correction to the degrees of freedom in the denominator, that is under discussion. d) *Q* ^2^_F3_ violates the total independence of the training and test sets or the corresponding validation parameters. e) We show on the graphs the robust median values of 500–1000 *Q* ^2^_F2_ values, that should be free of several disadvantages mentioned by other authors.

Before the discussion of our own work, we must make a remark on a special practice in the field of neural network research which also concerns validation. In the field of artificial neural network research,^[33]^ the terms are different. The term'hold out’ is used for a method, where they divide the sample to a training and a test set, then the parameters are optimized on the training set and test set is used only to tune the hyperparameters and assess the methods, but as everything is close to ready and all hyperparameters are determined, then the parameters of the final model are optimized on the merged data of the training and the test sets. The final merging of all data for the final model optimization is usually there in ANN applications, irrespective of the recipe used, e.g., repeated hold out, bootstrap, resampling, cross‐validation, *k*‐fold cross validation or parallel split to training, validation and test sets.^[33]^ This final merging of all data is encouraged as there is usually a lack of sufficient amount of data in most of the ANN studies. In our study, we never merged the test and the training sets in accordance with the OECD guidance.

## 2 Details of the Calculations

### 2.1 Datasets

We performed our calculations on datasets accessible in open repositories. Most of these are related to QSAR and they are stored in a the QSAR databank repository.^[34]^ Two data sets are accessible at UCI machine learning repository and Kaggle,[^35^, ^36^] one of them is data on an electric power plant and the other one is related to material science. There is a combined meteorological‐air pollutant dataset from Budapest in 2007 (AIR), the data are collected by prof. Imre Salma. These latter three datasets, unlike the other ones, are not related to QSAR. OECD principles have significance beyond QSAR modelling, especially principles 2, 4 and 5 are general. Therefore, we show our results also on the latter three datasets in order to go slightly beyond QSAR. The datasets are summarized in Table 1. We needed datasets as large as possible in order to investigate the sample size dependence, therefore, we merged the sets of the original studies, if there were any subsets. The details of the predictor and response variables can be found in the original articles and at the description pages of the repositories. In the case of the unpublished AIR dataset, the response variables were the daily air‐pollutant concentrations. The predictor variables were the meteorological data from the actual day, the air‐pollutant concentrations of the last two days and some calendar data to keep account of weekdays and months. The categoric data were one‐hot encoded. In the case of the MLR, ANN, and SVR models always a single response variable was used in the models. In the case of PLS2, a single model was built for all response variables. We did not use, assess, or interpret the models, their parameters and the experimental conditions of the original articles. We applied the datasets as numerical data on which we built new models after random resampling for a given sample size.

**Table 1:** Datasets. M=MLR, P=PLS2, A=ANN and S=SVR in modelling.

ID	Number of independent variables	Number of observations	Range of sampling	Descriptors	Response	Modell type	Ref.
**AIR y1‐y6**	(6 dep.) 38	363	30–350	calendar, pollution, meteorological	air pollutant concentrations	P	see text
**CE**	15	248	100–250	general/molecular parameters	cetane number	A, S	[34,37]
**CO y1‐y3**	(3 dep.) 7	103	30–90	composition, aging	concrete compressive strength, slump, slurry	P	[35,38]
**COC**	8	1030	30–500	composition and aging	concrete compressive strength	M	[35,36,39]
**DEG**	4	460	30–400	molecular parameters	degradation by OH radicals	M	[34,40]
**FBA**	9	632	30–500	molecular parameters	biotransformation half lives in fish	M	[34,41,42]
**FBB**	5	627	30–500	molecular parameters	bioconcentration factor in fish	M	[34,43]
**FPGD**	13	631	125–375	molecular parameters	flash point	A, S	[34,37]
**FPMD**	12	631	125–375	general parameters	flash point	A, S	[34,37]
**LFL**	6	1169	30–1000	molecular parameters	lower flammability limit temperature	M	[34,44]
**PP**		9568	30–1000	operating conditions of a combined cycle powerplant	electric power output	M	[35,36,45]
**SSA**	4	643		molecular parameters	soil sorption coefficient	M	[34,41,46]
**SSB**	4	643	30–500	molecular parameters	soil sorption coefficient	M	[34,41,46]
**TF TB**	(2dep.) 26	400	30–350	molecular parameters	boiling point flash point	P, A, S	[34,47]
**TOXA**	2	501	30–400	molecular parameters	tetrahymena toxicity	M	[34,48]
**TOXB**	6	449	30–400	molecular parameters	fathead minnow toxicity	M	[34,49]
**TOXC TOXD TOXE**	3	404	30–200	molecular parameters	algal toxicity	M M M	[34,50,51]
**TOXF**	23	566	100–500	general parameters	fathead minnow toxicity	A, S	[34,52]
**TOXG**	28	566	100–500	molecular parameters	fathead minnow toxicity	A, S	[34,52]
**UFL**	5	865	30–750	molecular parameters	upper flammability limit temperature	M	[34, 53]

### 2.2 Model Types

#### 2.2.1 MLR

We use the MLR abbreviation for the multivariate linear regression using ordinary least‐squares optimization. Some of the results were obtained in our previous study.^[19]^ In the case of MLR, unweighted least‐squares regression was applied and we used all of the predictor variables.

#### 2.2.2 PLS2

The PLS2 abbreviation refers to the partial least‐squared regression with more than a single response variable. It means, latent variables are formed both in the predictor and in the response spaces. PLS is an iterative method, where at first the predictor‐response latent vector pairs are identified sequentially using a maximal covariance criterion and least‐squares regression is used to determine the corresponding regression parameters. The further latent variable pairs are searched using the residual predictor and response matrices. The most important question is the number of the latent vectors. At first, we focused on the generalization of the repeated double cross validation method of Filzmoser et al.^[54]^ from single response to multi‐response cases. Unfortunately, the method using the Parsimony factor did not determine uniquely the number of the latent variable pairs for the multi response cases. Finally, we selected the number of latent variables via a compromise in validation parameters obtained in internal and external ways. This choice was different than the use of *R* ^2^ suggested by OECD. We checked the effect of standardization. We found that the performance of the models was better, if both predictor and response matrices were standardized in the cases, when the scale of the variables were different. Usually, it significantly reduced the number of latent variables and helped to obtain similar convergence feature of validation parameters with respect to the number of latent variables. Some examples of our investigation on the criterion of Filzmoser et al., on the effect of standardization and on our decision on the number of latent variables can be found in the supplementary material Figures S1, S2, S3 and in [55].

#### 2.2.3 ANN

Artificial neural networks are efficient non‐linear tools to perform classification or regression. Here we focus on simple structured ANN with one input, one hidden and one output layers, when the method can be interpreted as a nonlinear function approximation. Our models do not enter the field of deep learning. Partly, our datasets are not large enough to be used in deep learning studies and we would like to limit the size of our study on the simplest ANN cases without going into the details of sophisticated deep learning architectures. There is a large number of hyperparameters already for single hidden layer structures. The first hyperparameter is the number of neurons in the hidden layer. It was not easy to find a rule of thumb using validation parameters, since there was not any optimal choice between the different neuron sized models comparing their cross‐validation or external test performances. In our investigation we focused on the sample‐size dependence of the validation parameters, therefore, we were not able to select the number of neurons only with respect to the performance on the entire dataset. Finally, we fixed the number of the neurons in a way to have around as many weights to optimize as there were independent cases in the datasets at the smallest sample size used. We slightly modified this, if the proposed model was larger or smaller than the original model for the given dataset in the literature. The second hyperparameter is the choice of the activation function. We used logistic, tangent hyperbolic and relu ones. The choice of the optimizer also effects the performance of the models. We used the LBFGS (limited‐memory Broyden – Fletcher – Goldfarb ‐ Shanno algorithm), Adam (a first‐order gradient‐based optimization of stochastic objective functions, based on adaptive estimates of lower‐order moments^[56]^) and the SGD (stochastic gradient descent^[57]^) methods implemented in the Python scikit‐learn library.^[57]^ The common hyperparameter of the optimizers is the tolerance limit for the iterations, while there are several further hyperparameters for each of them (1,5‐9,3‐6 with respect to the three methods). Depending on the resolution of the values, there is a huge number of combinations for these hyperparameters even on a single dataset. This means, in the case of a given dataset we usually perform the optimization of the weights on the order of 10^6^. (In details: Adam 23328 models/dataset, SGD 6480 models/dataset, LBFGS 12 models/dataset; for each hyperparameter combination 150 optimizations of the weights for calculating *Q* ^2^_F2_ (5 and 10 times more for 5 and 10 fold cross validations, even if the number of the neurons in the hidden layer was fixed previously.) These hyperparameter scans seemed to be mandatory because we always found models with significantly better performance than those of the ones with default hyperparameters. Some aspects of the grid search and the choice of hyperparameters are detailed in the supplementary material in Figures S4 and S5. We should mention, that in the hyperparameter optimization we simultaneously used the validation parameters obtained on the external test set and the *Q* ^2^_LMO_ values of 5–10 fold cross validation. As it is detailed in the introduction, the QSAR nomenclature is slightly different than those of the neural network community. In the supplementary material Figure S6 we discuss, that both sets seemed to be adequate ones in our case.

#### 2.2.4 SVR

As 4^th^ modelling method we chose support vector regression. The performance of the method seems to be similar to ANN and different regression tree ones in the literature. We used the method with Gaussian radial basis function as kernel. There are some hyperparameters of the method, which of we performed grid search over kernel coefficient, epsilon parameter, regularisation parameter, shrinking, stopping and tolerance (Figure S7).

We mentioned, that we did not control directly the number of the support vectors in the models, we let its determination by the epsilon‐support vector regression implementation [58]. Our grid search over the hyperparameters concerned this epsilon parameter and it had some effect on the number of the support vectors. If we used a fix epsilon, the number of the vectors were able to change both in percentage with respect to the sample size and in nominal values. Usually we obtained models, where the number of the support vectors were around the 50–60 % of the sample size, depending strongly on epsilon. In the case of randomization, where there is no possibility of generalization of any model, the number of the support vectors approaches the number of cases.

### 2.3 Calculations

The calculations were performed with codes in R and Python developed by us. We intensively used the *pls* package in R and *scikit‐learn libraries* in Python.[^57^, ^59^]

The hyperparameters were separately optimized for each dataset. The selected data were divided into training and test sets with 80/20 ratio. Depending on the model type (PLS2, ANN, SVR) and the data set, 150–1000 repetitions were performed for each combination of the hyperparameters. The training set was used in the internal validation (goodness of fit, robustness). The test set was an external one to assess predictivity. In the case of PLS2, the number of the latent variables was determined for each dataset. In the case of the ANN and SVR models, 5–25 reasonable hyperparameter combinations were selected to be used in the sample‐size dependence calculations for each dataset. In some cases, M refers to the model numbers in the figures.

The sample size series were determined according to the number of available cases for each dataset. The smallest sample sizes were mostly fixed to be larger than the number of parameters in the optimization process. The models were underdetermined in a few cases of ANN and SVR, here the number of weights at the smallest samples was slightly larger than the number of cases. 500–1000 sample sets were randomly selected for each sample size of a given dataset. The sets were divided into training and test sets with 80/20 ratio. Repetition was not allowed within one set.

The leave many out cross‐validation was performed with random subdivision of the training data into *m*‐folds. Each sample set was divided into folds only once and for a given model the cross validation was not repeated with another subdivision.

The dependence of the validation parameters on chance correlation was calculated separately for the predictor and the response variables. We used *y*‐scrambling, where the response variables were reassigned to other cases randomly. In the case of *y*‐randomization, we generated random *y* responses from the distribution of the true responses. In the case of *x*‐randomization, the elements of the predictor matrix were randomly generated from distributions corresponding to the given original *x* variable vector.

The definitions of the calculated validation parameters are shown in Table 2. The goodness of fit was assessed by *R* ^2^ and *RMSE*. Robustness was quantified by *Q* ^2^_LOO_, *Q* ^2^_LMO_ and *RMSE*_LOO_. The latter was calculated by leave‐one‐out cross‐validation. The predictivity was measured by the *Q* ^2^_F2_ metric. It seems to be the most popular intensive validation parameter (external) from the *Q* ^2^_F1_‐*Q* ^2^_F3_ set. *RMSE*_test_ was calculated on the external test set, as well. In the case of the MLR models, non‐zero intercepts were allowed, therefore 0<=*R* ^2^<=1, *Q* ^2^_LOO_<=*R* ^2^ and *Q* ^2^_F2_<=1 were the limits. In the case of the other models, *R* ^2^, *Q* ^2^_LOO_, *Q* ^2^_F2_<=1 was the only theoretical limit and all intensive validation parameters might be negative, as well [19]. The figures in the next sections show the median of the validation parameters calculated on the 500–10000 sample sets at a given size. The trends were similar for the medians and the means of data, except a case, where it will be mentioned in the discussion.

**Table 2:** Validation parameters and notation.

Notation	Definition
*n*, *n*_test_, *n*_train_	number of cases in the sample, in the test and training sets
*p*	number of model parameters (without the intercept [60])
*y*_i_, $\bar{y}$	the *i*‐th and the average experimental response
${\hat{y}}_{i},\hat{y}$	the *i*‐th and the average modelled response
${\hat{y}}_{i/i}$ , ${\hat{y}}_{i/j}$	a cross‐validated response: leave‐one‐out and leave‐many‐out cases
*RSS*, residual sum of squares	$\sum_{i=1\&clineb}^{n}{\left(y_{i}-{\hat{y}}_{i}\right)}^{2}$
*MSS*, model sum of squares	$\sum_{i=1\&clineb}^{n}{\left({\hat{y}}_{i}-\hat{y}\right)}^{2}$
*TSS*, total sum of squares	$\sum_{i=1\&clineb}^{n}{\left(y_{i}-\bar{y}\right)}^{2}$
*PRESS*, cross validated residual sum of squares	$\sum_{i=1\&clineb}^{n}{\left(y_{i}-{\hat{y}}_{i/i}\right)}^{2}$
$R^{2}$ , coefficient of determination	$1-\frac{RSS}{TSS}$
*RMSE*, root mean squared error	$\sqrt{\frac{RSS}{n}}$
$Q_{LOO}^{2}$ , leave‐one‐out cross validation *R* ^2^	$1-\frac{PRESS}{TSS}$
$Q_{LMO}^{2},$ leave *M* out cross validation *R* ^2^, *j* is the model where the *i*‐th data is not used	$1-\frac{\sum_{i=1}^{n}{\left(y_{i}-{\hat{y}}_{i/j}\right)}^{2}}{TSS}$
*RMSE_LOO_*	$\sqrt{\frac{PRESS}{n}}$
$Q_{F2}^{2}$ , ($R^{2}$ for test set) [22]	$1-\frac{\sum_{i=1}^{n_{\mathrm{test}}}{\left(y_{i}-{\hat{y}}_{i}\right)}^{2}}{\sum_{i=1}^{n_{test}}{\left(y_{i}-{\bar{y}}_{test}\right)}^{2}}$
	
*RMSE*_test_	as *RMSE* on test set

The correlations among the different validation parameters are calculated not between the validation parameters, but between their respective ranks in order to be less sensitive to nonlinear effects. In this way, the rank correlations emphasize the monotonous relationship between two validation parameters and not necessarily a linear one.

## 3 Results and Discussion

The aim of this manuscript is to show the common and the different features of the model types with respect to the OECD 4^th^ principle. To obtain these, we mostly show the trends with respect to the sample size.

**Figure 1:**
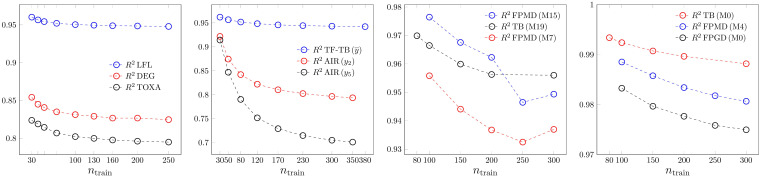
Goodness of fit. *R* ^2^ dependence on the sample size. From left to right: MLR, PLS2, ANN and SVR.

The most common measure of goodness of fit is the coefficient of determination. *R* ^2^ is shown for the four modelling methods in Figure 1. Results of three models are shown on the graphs. In the case of ANN, two models belong to the same dataset. In the PLS2 case, two variables of the AIR dataset and an average result of the TF‐TB datasets are shown. The trends are strictly monotonically decreasing in all cases, except two of the ANN models. In the case of ANN, mostly we got clear trends, but we obtained a minimum for FPMD model 7 and 15 (M7, M15 on the graph). The higher *R* ^2^ values at small sample sizes reflect the effect of possible overfitting. This is an artifact that may disturb any simple conclusion based on the magnitude of a single validation parameter. No models trained on smaller sample sizes should be preferred just because of higher values for such a metric with false trend. We emphasize the scale on the ANN and especially on the SVR case. These models have a very high flexibility to reproduce many kinds of data, but as we see later, this flexibility need not cause robustness and predictivity with similarly high measures. We got smaller *R* ^2^‐s only for the badly modellable TOXF and TOXG datasets. In the case of SVR, the extreme goodness of fit is partly the result of the increased number of support vectors.

**Figure 2:**
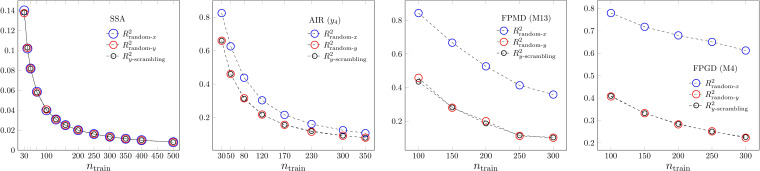
Chance correlation. *R* ^2^ of randomized data. From left to right: MLR, PLS2, ANN and SVR.

This extraordinary flexibility can be seen in Figure 2, where we show the results of different randomization processes to estimate chance correlation. In the case of MLR, random responses of all sorts were weakly modellable. PLS2 was able to model random responses as long as the number of parameters was high with respect to the number of cases to be reproduced. Apparently for small sample sizes, the search for maximal covariances is able to provide latent predictor and response variables that show a high degree of chance correlation even with random responses. Both in MLR and PLS2 the ability for modelling random responses decays exponentially with increasing sample size. On the contrary, the decay is slow and resembles a linear trend in the case of ANN and SVR. In the case of ANN, reasonably efficient fitting of random responses is still possible even at medium sample sizes. In the case of SVR, the model is able to reproduce (goodness of fit is shown here) or to find any hidden a chance correlation, if *x* was randomized. We obtained *R* ^2^‐s over 0.9 for chance correlation in many SVR cases. Since this high level of chance correlation does not seem to be really present in the randomized data, we might say that these very flexible techniques might create chance correlation, if the number of the support vectors approaches the sample size (cf. Figure S7). The other difference between MLR and the other types of modelling methods is the behaviour with respect to *y* and *x* randomizations. In the case of MLR, the three methods provide similar results. The computationally simplest *y*‐scrambling (shuffling) is as effective as the two other random methods. In the case of mean values, they are identical, in the case of median values, there was a hardly detectable difference between the two random value generation and scrambling. In the case of the other three model types, there are significantly different *R* ^2^‐s on predictor and response randomized data. In our PLS2 datasets, we have always more predictor variables than responses. We think, that it is slightly easier to find some detectable chance correlation among more variables, when the *x* latent vectors are used. In the case of less variables, as in *y*‐randomization, the possibility of finding a highly correlated latent *y* vector to existing internally correlated *x* vectors is less probable. In the case of ANN, we always find a substantial difference for *x* and *y* randomizations. Maybe, again the difference between the number of *x* variables and the single *y* variable is one reason, but it might go to the enormous flexibility provided by ANN, partly by the large number of weights and possibility of non‐linear function fit feature of ANN. In the case of SVR, the close to one *R* ^2^ of randomized *x*‐data clearly shows the incredible classification/regression power of the support vector systems for many datasets. The correlation within the predictor variables seems to be crucial. In the case of *x* randomization, both the inter‐predictor and the predictor‐response correlations are removed. Here the task is to model totally random data. If *y* is shuffled or randomized, the remaining correlations in the predictor variables contradict to the random *y*‐s. This contradiction results in a weaker correlation between predictor‐response data pairs and thus worse reproduction of the training responses.

These results on “created“ chance correlations at small sample sizes or at *x* randomization support the idea, that goodness‐of‐fit validation at high performance methods, like ANN or SVR, might have only a restricted relevance contrary to the OECD 4^th^ principle and guidance. Since model performance is generally assessed via the estimation power of the response variables, we think that *y*‐randomization and *y*‐scrambling are the adequate ways to measure chance correlation, if there are reasonable inter‐predictor correlations. In the case of successful MLR models, inter‐predictor correlations are negligible. The choice between *y* and *x* randomizations is optional. In the other cases, the random *x* approach totally destroys the data structure and creates a uniform variable space distribution independently from the original data. In the case of normalized data, it creates a spherical data cloud in Euclidean variable space, destroys the difference between Mahalanobis and Euclidean variable spaces. The data become feature‐ and shapeless up to a level, where it is not an adequate reference, when we are interested in the estimation power on *y*‐s. Our conclusion is to use randomization only on *y* and to be effective do it by scrambling. The *RMSE*‐like randomization values are shown in Figure S8.

The second aspect of validation in the OECD guidance is robustness. It is defined as a part of internal validation, where several models are built on a part of the training set or on resampled parts of the training set. The most common method is cross validation, but it can be performed by properly designed bootstrap, as well. The simplest cross validation is the leave‐one‐out approach, where data are omitted one by one from the model building and the calculated response of the omitted case is used in the calculation of the validation parameter. It is an internal method, because the final model is always the one, where all cases of the training set are used in the parameter optimization.

**Figure 3:**
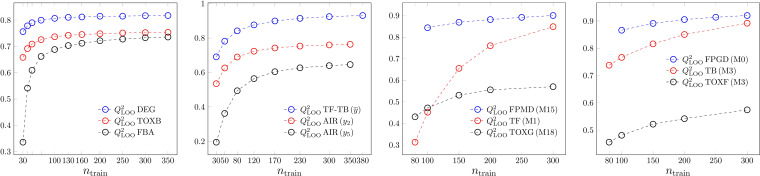
Robustness. *Q* ^2^_LOO_ dependence on sample size. From left to right: MLR, PLS2, ANN and SVR.

The sample size dependence of *Q* ^2^_LOO_‐s is shown in Figure 3. Contrary to *R* ^2^, there is no artifact here for small sample sizes, the robustness of the models is weaker for models derived on small training sets. Theoretically, the infinite sample size limit of *Q* ^2^_LOO_ is *R* ^2^,[^8^, ^12^] but the number of cases in our data sets was not large enough to reach this limit. This limit is close to unreachable at our sample sizes due to the high flexibility of ANN and SVR. The goodness‐of‐fit and the robustness curve limits are approached, if the model performance is overall excellent.

**Figure 4:**
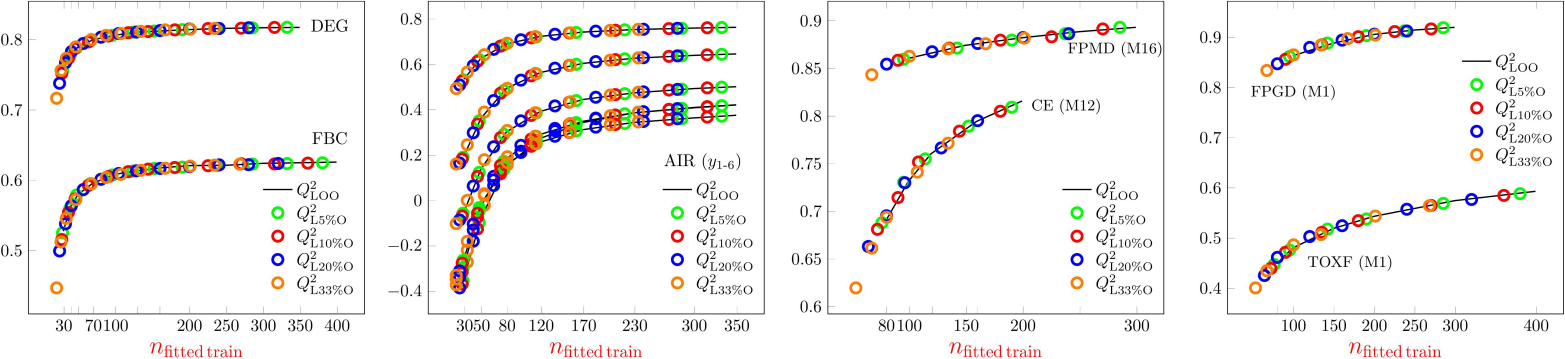
Robustness. Scaling of *Q* ^2^_LOO_ and *Q* ^2^_LMO_, if the data are shown with respect to *n*_fitted_. From left to right: MLR, PLS2, ANN and SVR.

We show in our preliminary study,^[19]^ that leave‐one‐out and *m*‐fold cross validations can be rescaled to each other, if we plot the results with respect to the number of the cases used in the fit during the cross‐validation model building (*n*_fitted_) instead of the training set size (*n*_train_). We checked it for the other model types, and we found that in all the 4 types of models the different *m*‐fold *Q* ^2^_LMO_ values are very close to the *Q* ^2^_LOO_ curve, if *n*_fitted_ is used on the abscissa (Figure 4). The same scaling of the *RMSE*‐like data can be seen in Figure S9. In the case of linear models, *Q* ^2^_LOO_ can be calculated without performing extra determination of the model parameters, the corresponding hat matrix elements can be used to calculate PRESS (Table 2). In the case of ANN and SVR one needs as many new optimizations as cross‐validated models one would like to have. Computationally this means, that for the linear models the leave‐one‐out scheme is preferred, while the *m*‐fold versions for ANN and SVR. Our results suggest that it is always enough to calculate only the computationally feasible cross validation, because the results might be scaled to each other. Of course, not only the behaviour of the median values, but also the possible range (uncertainty) of the validation parameters is important. It can be seen in the supplementary material (Figure S10), that we need not withdraw our conclusion taking into account this aspect. The use of leave‐one‐out scheme instead of the leave‐many‐out one in the linear models and the use of leave‐many‐out scheme instead of the leave‐one‐out one for ANN and SVR did not increase the uncertainty of the assessment. We should note here, that in experimental applications usually we have repetitions in the data, e.g., in analytical chemistry at least three measurements are performed on the same sample. We show in the supplementary material in Figure S11, how it changes our proposed scheme, if we use leave‐case‐out or leave‐sample‐out schemes. Anyway, our scheme works well, if we simply use the averages of the same sample measurements instead of the individual measurements.

**Figure 5:**
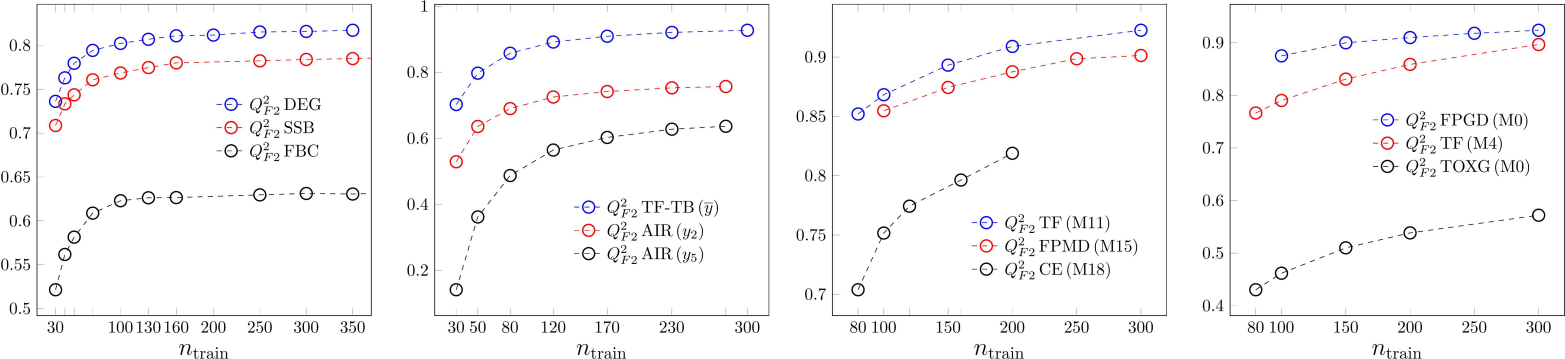
Prediction. *Q* ^2^_F2_ dependence on sample size. From left to right: MLR, PLS2, ANN and SVR.

The third validation element of QSAR models is external validation serving the purpose of assessing predictivity. External means, that a test set is created from the data that are not used in the optimization of the model parameters. This does not hold for the hyperparameter optimization. If there are more models, in the selection of the final one we might use the results on the independent test set. We show the *Q* ^2^_F2_ values in Figure 5. The trends are correct in a sense, that models developed on small training samples perform in average weaker than models optimized on large datasets. We can see that the performance of the ANN and SVR models do not converge in this sample size to the *R* ^2^ values of Figure 1. We performed calculations to check test/training splitting, If a Kennard‐Stone [61] like algorithm is used to do the split, the elements of a pair of repeated measurements are divided to both sets. We show in the supplementary material (Figure S12), how rhapsodically on different data sets it changes the trends, e.g. *Q* ^2^_F2_‐s becomes similar to the misleading *R* ^2^ ones. It means, one should know, that the use of Kennard‐Stone splitting highly bias the trends with respect to the simple random splitting and the validation parameters should be interpreted slightly differently.

As we mentioned, in the case of an optimal model we usually think that *R* ^2^, *Q* ^2^_LOO_ and *Q* ^2^_F2_ approximate each other quite well above a certain sample size. This behaviour is present for most MLR and PLS2 models. In the case of ANN or SVR, the limit of goodness of fit was always reasonable higher than the other ones. The values of *Q* ^2^_F2_ are higher in most cases than that of *Q* ^2^_LOO_ in our graphs, where the medians of 500–10000 models are shown. In some cases, the *Q* ^2^_F2_ and the *Q* ^2^_LOO_ curves are ordered oppositely, if averages are depicted. The difference between the two estimates of expected value (mean and median) is probably related to the presence of badly modelled outliers. This suggests to use the median, that is the robust measure. Our result that robustness in average is sometimes weaker than predictivity might be related to the so‐called masking effect. For examples concerning this paragraph we refer to the graphs in the supplementary material, Figure S13. We should mention, that in the case of some SVR data, we found some models whose behaviour was different from that of the other ones. At maximal sample sizes they provided as good *Q* ^2^_F2_‐s as the other ones, but at small sample sizes their predictivity decreased reasonably. The number of the support vectors were close to the maximum in these models, similarly as we saw in the randomization section. For the stable models the number of the support vectors was usually around the half of the maximum. Therefore, we propose to always check the number of support vectors as a warning signal in the case of SVR models (see again Figure S7)

**Figure 6:**
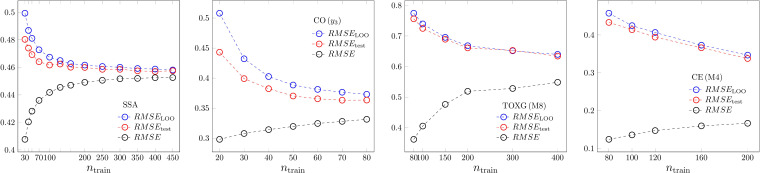
*RMSE*‐like validation parameters. From left to right: MLR, PLS2, ANN and SVR.

Up to now we have justified our results using intensive validation parameters in the manuscript, extensive parameters have been shown in the Supplementary material. Now, a set of examples is shown for the extensive parameters in Figure 6. Here, mostly we show only the sample size dependence of one model to avoid crowded graphs. We found that the same conclusions can be drawn based on the *RMSE* family of validation parameters as on the *R* ^2^ family. In the case of MLR one can see that the common limit of the three measures is reached. Such a limit does not exist within the sample size accessible in our datasets for PLS2, ANN and SVR. The weakest convergence is for SVR (cf. Figure 1–2) One can see, as well, that the goodness of fit *RMSE* misleadingly assess small sample models as apparently better than the large ones. Our findings on *x‐* and *y*‐randomization and on the scaling of leave‐one‐out and leave‐many‐out cross validations have been justified in the supplementary material for *RMSE*‐like validation parameters (Figures S8 and S9).

When we started our investigation on sample size dependence a few years ago, our idea was initiated by scientists who debated quite a lot around the feasibility of internal or external validations. We think, that one way to check the independent information content of the different validation parameters is their correlation for a set of given models. Since the most popular measure of correlation, the so‐called correlation coefficient is inherently designed to indicate the strength of a linear relationship between two vectors, we use rank correlation, that shows rather the monotonic relation between the two validation parameters. If we are interested in overall correlation of the three validation aspects, we might calculate the rank correlation over all models developed for all datasets, irrespective of the hyperparameters and the used sample size. Another approach is, if we use classes of the models, where a class is formed by the repeated set of models on a given dataset with the same hyperparameters and sample size.

The first idea is summarized in Table 3, where some of the rank correlation pairs are shown for datasets with different models. Here, the data are calculated among the six validation parameters (three intensive and three extensive ones).

**Table 3:** Rank correlations of all models for different model types.

	MLR	PLS2	ANN	SVR
intensive/extensive pairs				
*R* ^2^/*RMSE*	−0.93	−0.97	−0.99	−0.98
*Q* ^2^_LOO_/*RMSE*_LOO_	−0.95	−0.94	−0.99	−0.98
*Q* ^2^_F2_/*RMSE*_test_	−0.87	−0.85	−0.96	−0.95
goodness of fit/robustness				
*R* ^2^/*Q* ^2^_LOO_	0.90	0.71	0.34	0.38
*RMSE*/*RMSE*_LOO_	0.89	0.74	0.33	0.36
*R* ^2^/*RMSE*_LOO_		−0.71	−0.32	−0.35
*Q* ^2^_LOO_/*RMSE*	−0.83	−0.67	−0.32	−0.35
goodness of fit/predictivity				
*R* ^2^/*Q* ^2^_F2_	0.59		0.31	0.31
*RMSE*/*RMSE*_test_	0.65	0.59	0.32	0.30
*Q* ^2^_F2_/*RMSE*	−0.60	−0.55		−0.30
*R* ^2^/*RMSE*_test_	−0.65	−0.61	−0.32	−0.33
robustness/predictivity				
*Q* ^2^_LOO_/*Q* ^2^_F2_	0.65	0.76	0.74	0.69
*RMSE*_LOO_/*RMSE*_test_	0.68	0.74	0.74	0.66
*Q* ^2^_LOO_/*RMSE*_test_	−0.68	−0.75	−0.74	−0.68
*Q* ^2^_F2_/*RMSE*_LOO_	−0.66	−0.76	−0.74	−0.69

Since the response variables in the different datasets might have different magnitudes, the *RMSE*‐like validation parameters were calculated on standardized data. In the case of PLS2, ANN and SVR these were the settings anyway. In the case of MLR it did not change the results, only the magnitude of the *RMSE*‐s became comparable. If we check the data in Table 3, the intensive‐extensive pairs for the same aim (goodness of fit, robustness, prediction) are highly correlated. This means, that there are no significant differences, if we use only one of them. In the case of the rank correlations between the two aspects of internal validations, they are rather significant for all of the 4 pairs in the case of MLR (0.83–0.90) and they are still high for PLS2 (0.67–0.74). But these rank correlations are between 0.32 and 0.38 for ANN and SVR. Comparing the goodness of fit – predictivity pairs, the results show less correlation, 0.55–0.65 for MLR and PLS2 and 0.31–0.33 for ANN and SVR cases. On the contrary, the third pair (robustness‐predictivity) correlates better (0.65–0.74), specifically it is around 0.74 for ANN. Altogether, we found differences with respect to the methods. The two internal validation purposes correlate well for the linear models, implying that they are practically redundant. In the cases of ANN and SVR, the goodness of fit does not correlate to the others, but this is not because of the importance of goodness‐of‐fit measures of these methods because of the irrelevance of goodness‐of‐fit as a validation aspect for these methods (c.f. Figures 1 and 2 and their discussion). External validation seems to provide new information mostly, but in the case of ANN it is close enough to cross validation, implicating that it is enough to use one of them during, e.g., the hyperparameter tuning.

**Figure 7:**
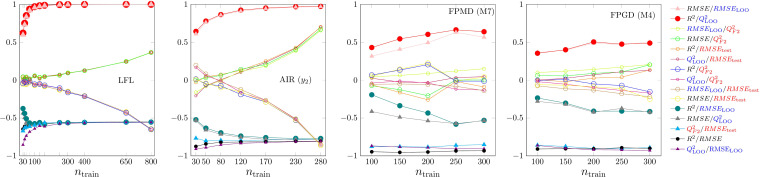
Sample size dependence of intra‐class rank correlations. From left to right: MLR, PLS2, ANN and SVR.

The second type of rank correlations is shown in Figure 7. In Figures 1, 2, 3, 4, 5, 6 we showed the median of a set of 500–10000 models built for one dataset using a given sample size and hyperparameters. The variability of the models is a result of the random selection of the sample (and in the case of ANN of the non‐unique results of the numerical optimization, as well). We call our correlations within a set intra‐class correlation. The models within a set are only partly independent of each other. If the sample size is close to the dataset size, the assignment of a given case to the training or to the test part is related to the so‐called allocation problem. It is known in the literature of design of experiment, that there are data allocations that provide reasonably better validation parameters for both training and test parts. Some aspects of this topic are summarized, e.g., in one of our previous studies [62].

The intra‐class rank correlations depend rather on model type and dataset (Figure 7). Each figure shows 15 rank correlation curves, three of which represent intensive‐extensive validation parameter pairs for the same aim, e.g., *R* ^2^ and *RMSE*. There is a more or less significant correlation for most of these pairs. In the case of MLR, the rank correlation is weaker than in the case of PLS2, and it is close to −1 for ANN and SVR. This means, that in the case of MLR we might use both intensive and extensive parameters, but in the case of ANN and SVR their information content is very similar within the intra‐class concept. The rank correlation parameters assessing goodness of fit and robustness clearly show an increasing trend and at large sample size close to perfect correlation for MLR and PLS2. This means, that at small sample sizes the two internal validations provide less dependent information, but over a sample size, depending on the dataset, one of them is superfluous. In the case of ANN and SVR the correlation is usually weaker, but we found a strong dataset and hyperparameter dependence even for the trends of the curves (Figure S14) It is not surprising, since we mentioned earlier, that goodness of fit provides very limited information on these models, while robustness (as defined in OECD) is one of the most popular aspect used for model selection, e.g., for ANN.^[7]^ The last group of rank correlations are the internal‐external ones. Here, our results are strictly reduced to our intra‐class relation and they are driven by the allocation problem. At small sample size the allocation of the training and the test parts are independent from each other and the correlation of internal and external validation parameters within a group of similar models almost vanishes. Increasing the sample size, there is a chance that the assignment of badly modellable and regular cases are exchanged in the training and test sets. Furthermore, in the case of the intensive parameters the range of the data in the two sets also has a large effect, as it highly affects the divisor in the sum of square term (see Table 2, [24, 62]). The overall effect of this is clearly visible in the enhanced negative rank correlations (Figure 7 and S14) and on the exchange in the quartiles of internal and external good parameters (Figure S15)‐

## 4 Conclusions

The aim of our study was to assess the QSAR‐OECD validation principle on types of modelling algorithms. The OECD 4^th^ principle defines three aspects of validation: internal validation for goodness of fit and robustness and external validation for predictivity. Herein, we extended our previous study on the sample size dependence of MLR models [19]. We modelled mostly QSAR data accessible in repositories by MLR, PLS2, ANN and SVR techniques. Most of our conclusions are drawn on the basis of the behaviour of different validation parameters with respect to sample size. As our conclusion, we summarize some rules of thumb on validation in Table 4 which were provided in our study.

**Table 4:** Proposed validation scheme. Validation parameters in bold are the suggested ones.

	Internal		External	Be careful
model	goodness of fit	robustness (with cross validation)	predictivity	
MLR	**R^2^** and/or **RMSE_training_**	not necessary at large sample size **Q^2^**_**LOO**_ (≈Q^2^_LMO_) and/or **RMSE_LOO_** (≈RMSE_LMO_)	**Q^2^**_**F2**_ and/or **RMSE_test_**	–
PLS2				to standardize x‐y at determination of the number of latent variables
ANN	maybe, R^2^ and/or RMSE_training_	**Q^2^**_**LMO**_ (≈Q^2^_LOO_) and/or **RMSE_LMO_** (≈RMSE_LOO_)		to standardize to optimize hyperparameters to check number of support vectors in SVR
SVR				

Similarly to the MLR case, the goodness‐of‐fit parameters (*R* ^2^, *RMSE*) misleadingly overestimate the models on small samples than on reasonable sample sizes, which is an understandable yet important example of the bias‐variance trade‐off. In the case of ANN and SVR (and at small sample sizes of PLS2) goodness of fit does not seem to be important, since these very flexible methods are able to reproduce training set data almost perfectly. This is valid also for randomized and scrambled data, ANN and SVR are able to find or more precisely to create chance correlation in random data, especially in the case of random predictor variables. This result was obtained, when we tested the sample size dependence of randomization methods. Additionally, in the case of MLR *x‐* and *y*‐random generation of data from the original data distributions are as effective as the simple shuffling of *y* values. The latter is proposed to be used as the numerically simplest solution. In the case of PLS2, ANN and SVR the predictor and the response randomizations are different, the methods are able to find more chance correlation, or they are able to create an artifact of chance correlation. In the SVR case, modelling might be so effective, that the goodness of fit *R* ^2^ is close to one for several *x*‐randomized data, if the number of the support vectors is not limited.

We checked via the sample size dependence, that our rescaling law proposed for LMO metrics in the MLR article is valid for PLS2, ANN and SVR. We found, that the leave‐many‐out cross‐validation parameters can be mapped on the leave‐one‐out curves by simply using the number of fitted data in the cross‐validation process instead of the number of cases in the total training set. This means, that the computationally cheaper versions should be used, leave‐one‐out cross validation for MLR and PLS and leave‐many‐out schemes for ANN and SVR. This finding remained justified, when we additionally checked the variance (range) of the individual *Q* ^2^_LOO_ and *Q* ^2^_LMO_ parameters, where we were not able to differentiate between *Q* ^2^_LOO_ and *Q* ^2^_LMO_ according to their statistical uncertainty.

We assessed the interdependence of the different validation parameters by calculating their rank correlations for the models. We performed this in two ways. In general, we merged the results of all models on all datasets and calculated the rank correlations between the validation parameters. In another approach, the rank correlations are calculated on subgroups of models built on the same sample size with the same hyperparameters. The latter we call as intra‐class rank correlation and here it was possible to check the sample size dependence. Results obtained by this second method were also related to the so‐called allocation problem as the sample size approximated the dataset size. We found that goodness of fit and robustness correlate quite well over a sample size for MLR and PLS2. This means that for reasonably large models one of the two validation aspects is redundant. In the correlation of internal and external validation parameters, we found that the assignment of good and bad modellable data to the training or the test causes negative correlations in accordance with our previous results on allocation.^[62]^

We checked the validity of our findings related to cross validation parameters, if there are repeated measurements in the data. We found that cross validation with simple random selection of cases upsets the trends and provides unfeasible validation parameters. The *Q* ^2^_LOO_ or *Q* ^2^_F2_ parameters changed their trends similar to the misleading *R* ^2^ one. We propose to use cross validation with leaving out all measurements related to a sample or to use their averages. We found that Kennard‐Stone test/training splitting drastically effects the trends with respect to random splitting. The changes are rather accidental for the different data sets.

## Conflict of interest

None declared.

## Supporting information

As a service to our authors and readers, this journal provides supporting information supplied by the authors. Such materials are peer reviewed and may be re‐organized for online delivery, but are not copy‐edited or typeset. Technical support issues arising from supporting information (other than missing files) should be addressed to the authors.

Supporting Information
