# Supplementary material for: The Relevance of Goodness‐of‐fit, Robustness and Prediction Validation Categories of OECD‐QSAR Principles with Respect to Sample Size and Model Type
Source: Mol Inform. 2022 Jul 25;41(11):2200072. doi: 10.1002/minf.202200072 (PMC9787734; doi:10.1002/minf.202200072)
Supplement: Supplementary file 1 — Supporting Information [file MINF-41-2200072-s001.pdf]

molecular informatics

Supporting Information

The Relevance of Goodness-of-fit, Robustness and Prediction Validation Categories of OECD-QSAR Principles with Respect to Sample Size and Model Type

Péter Király, Ramóna Kiss, Dániel Kovács, Amine Ballaj, and Gergely Tóth*© 2022 The Authors. Molecular Informatics published by Wiley-VCH GmbH. This is an open access article under the terms of the Creative Commons Attribution License, which permits use, distribution and reproduction in any medium, provided the original work is properly cited.

Supplementary material for

The relevance of goodness-of-fit, robustness and prediction validation categories of OECD-QSAR principles with respect to sample size and model type

Péter Király, Ramóna Kiss, Dániel Kovács, Amine Ballaj and Gergely Tóth*

Institute of Chemistry, Loránd Eötvös University, Pázmány S. 1/A, 1117 Budapest, Hungary

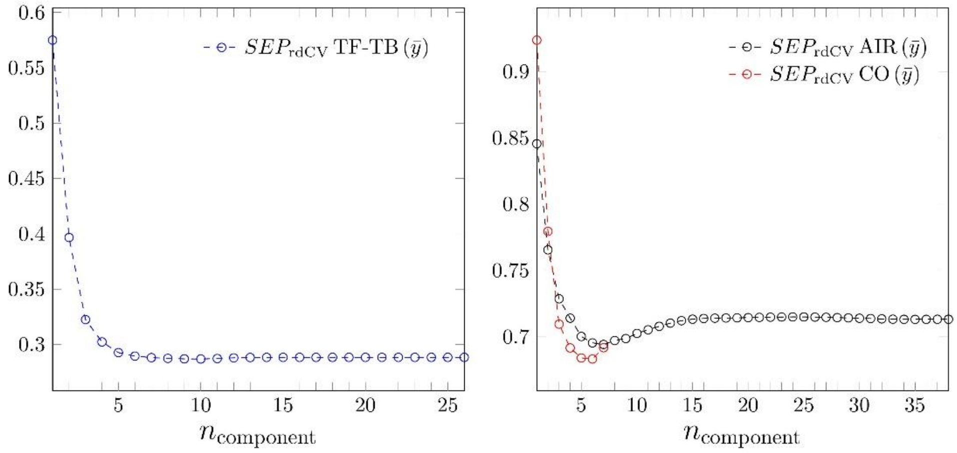

Figure S1. *SEP* values of repeated double cross validation (rdCV) as function of the PLS2 modelling hyperparameter in case of the mean response variable. Figure on the left shows a minimum plateau for the TF-TB dataset on the contrary the other two PLS2 modelled datasets (right) had a clear minimum in the *SEP* curve. Neither of the minima coincided with the rdCV optimum number of components using the default parsimony factor. The error curves show the same trends as their R^2-like counterparts in Figure S2.

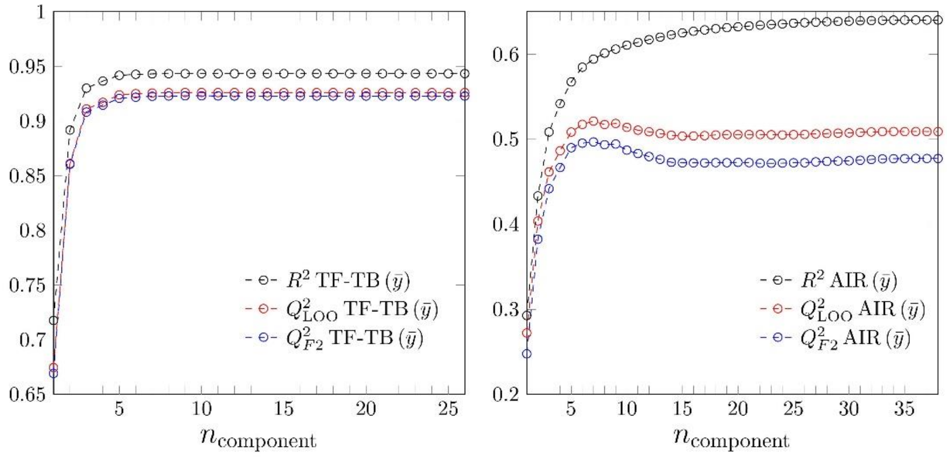

Figure S2. R^2-like validation parameters as function of the PLS2 modelling hyperparameter in case of the mean response variable. Figure on the left shows that the goodness-of-fit, robustness and predictivity curves reaching a plateau when the number of components equals 4 (TF-TB dataset) (this value was used in the models used in the manuscript). On the right: in contrast one can see that the robustness and predictivity curves for the AIR

dataset show a clear maximum at $n_{\text{component}}$ = 7, despite this feature we chose a higher value for modelling based on the individual goodness-of-fit curves (cf. Figures S1 and S3).

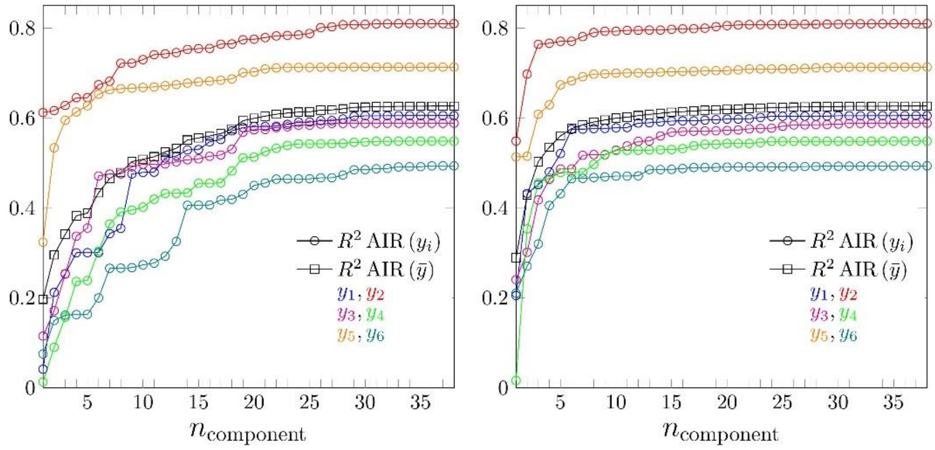

Figure S3. Effect of standardization for PLS2 modelling on goodness of fit as a function of the number of components (latent variables) for individual and model mean response variables (AIR dataset). On the left: predictor and response matrices are not standardized previously to modelling. On the right: both matrices were standardized. By standardization of both matrices the individual R^2 curves reach a plateau at much lower number of components which enables a chance to model the dataset by a simpler model on contrary to non-standardized dataset models. The number of latent variables for the AIR dataset was chosen in accordance with the individual R^2 curves to be 10 where most curves reach a plateau.

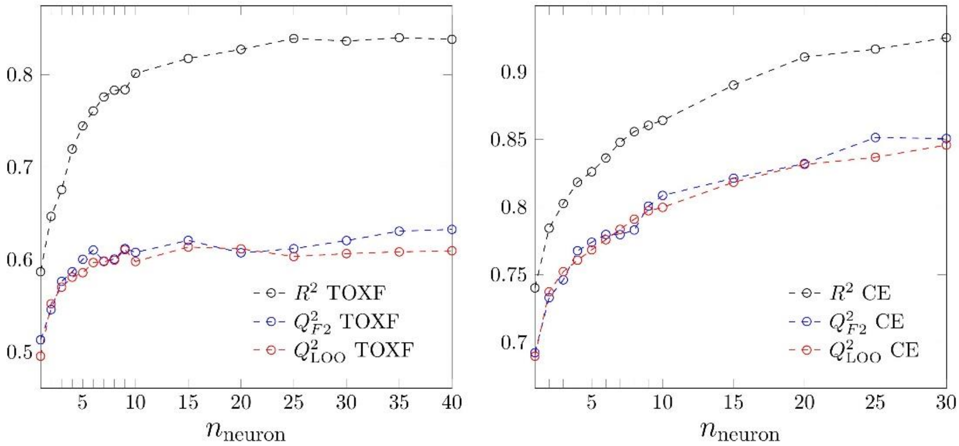

Figure S4. R^2-like validation parameters as a function of the number of neurons. Initial exploration of the hyperparameter space of the neural network included testing the different validation categories on their neuron number dependence (the hyperparameters solver type, optimization tolerance were modified, others were kept at default values). In the left figure a plateau type dependence is depicted while for the CE dataset (right) typical monotonically increasing curves are shown, besides these types depending on the data set one can register also overfitting with increasing number of neurons (not shown here). The unclear trends lead to the choice of neuron numbers as detailed in the article.

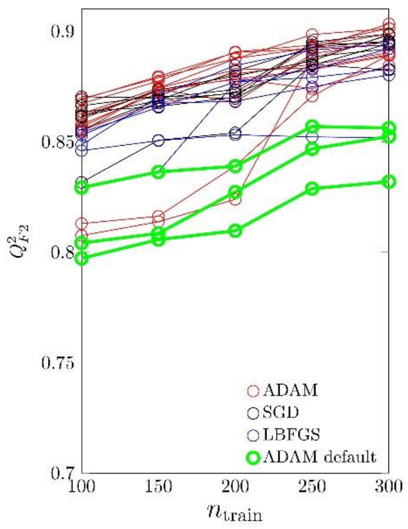

Figure S5. Necessity of tuning the hyperparameters of the neural network. The green curves show the training size dependence of predictivity of the default parameterized neural networks with ADAM solver with different types of activation functions. Tuning the solver parameters results more predictive neural networks (red curves). Comparing the quasi-newton LBFGS and the stochastic solvers (ADAM, SGD) one can say that usually the stochastic ones are more predictive than the former one.

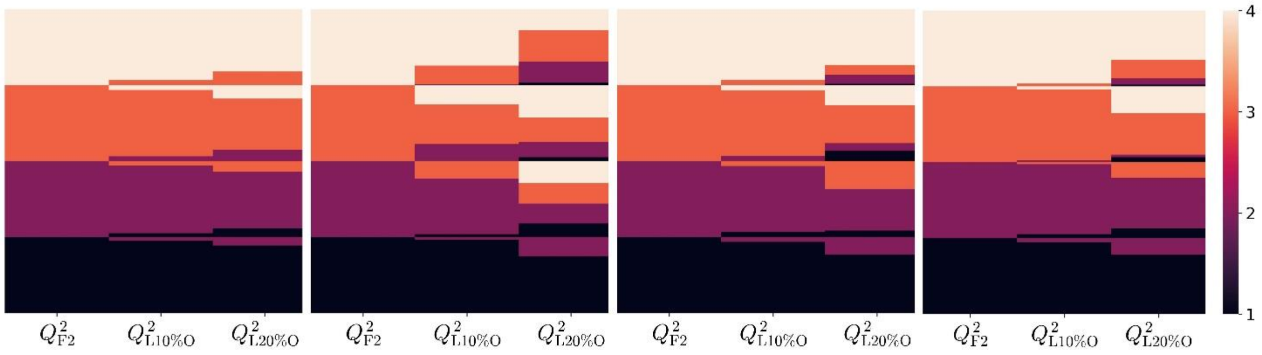

Figure S6. The hyperparameter scan of the neural networks was quantified with the external coefficient of determination (Q^2_{F2}) and the internal 10-fold and 5-fold cross validation ($Q^2_{\text{L10\%O}}$, $Q^2_{\text{L20\%O}}$) to check whether good predictivity coincides with good robustness. For example, figure left and left middle show the performance of neural networks with ADAM optimizer, logistic activation function and an optimization tolerance of 10^{-4} with initial learning rates 10^{-1} and 10^{-2} on the FPMD dataset (other parameters were varied on a broad range). The Q^2_{F2} range of the obtained models was split at their 1st, 2nd and 3rd quartiles and the resulting group belonging color-coded. The corresponding robustness values were also split on their quartile values and sorted within the group. One can see in figures left and left middle that there is a good correspondence between predictivity and robustness of neural networks which is lowered when the initial learning rate is decreased. Figure right middle depicts performance of SGD models with constant and adaptive learning rates together, figure right shows SGD models with inverse scaling learning rate.

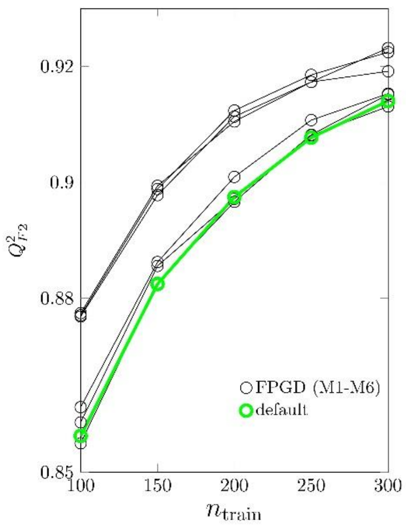

Figure S7. On contrary to neural networks the default hyperparameter set (green curve) for the rbf-kernel SVR showed only small difference in predictivity compared to tuned ones (black curves) as a function of training size. The curves show the result for the FPGD dataset, for this dataset the models which used a lower number of support vectors than the training size had better predictivity.

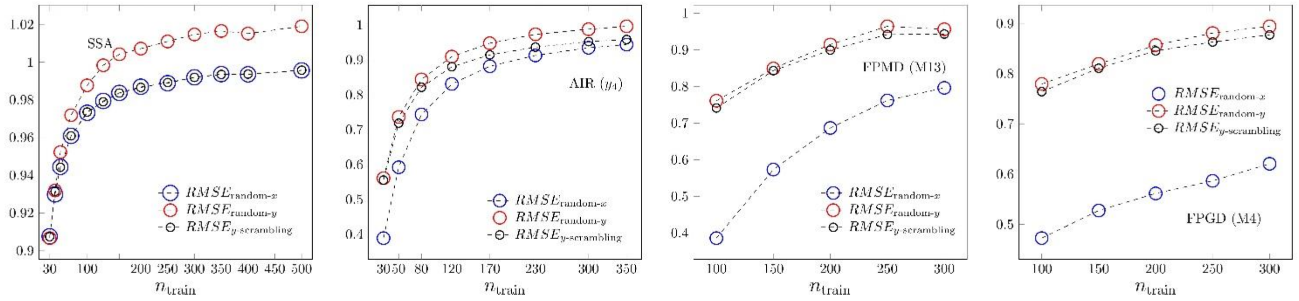

Figure S8. Accounting for chance correlation with the *RMSE*. Compared to the results of the R^2 variants we notice that for MLR (left) *x*-randomization and *y*-scrambling have a common trend, for larger sample sizes the *y*-randomization curve runs above the former ones. PLS2 (middle left) trends are also different the *y*-scrambling curve is delimited by the randomization curves for all training sizes. For ANN (middle right) and SVR (right) behaviour we notice a small difference between *y*-scrambling and *y*-randomization in contrary to the corresponding R^2 curves (cf. Figure 2).

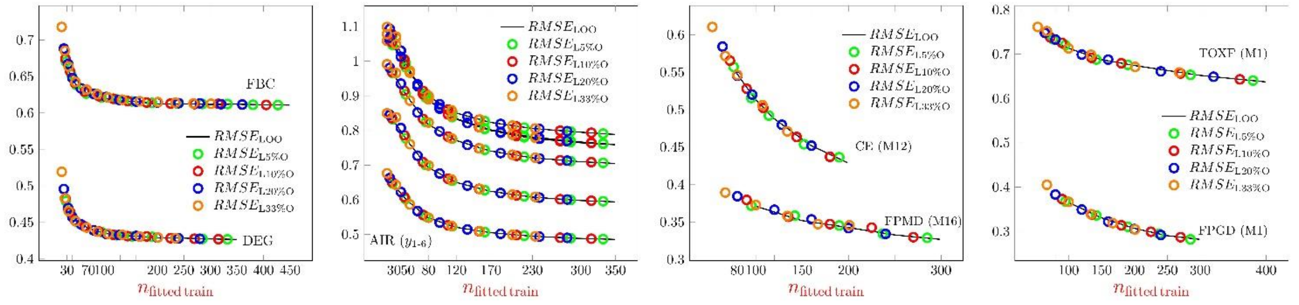

Figure S9. Since Q^2 $_{\mathrm{LOO/LMO}}$ and $RMSE_{\mathrm{LOO/LMO}}$ variants are connected by the *PRESS*, the LOO/LMO scaling rule is also valid for MLR (left), PLS2 (middle left), ANN (middle right) and SVR (right) models.

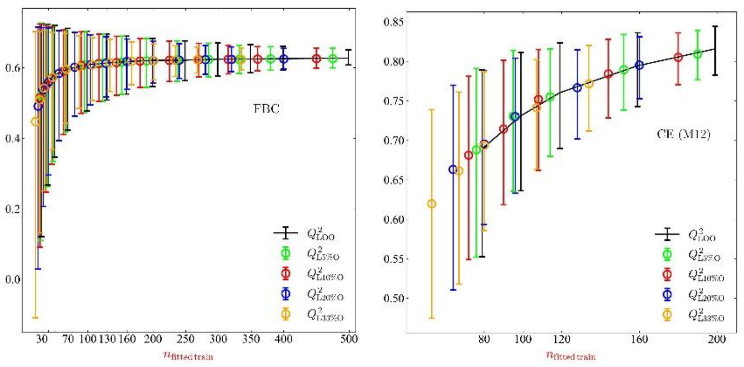

Figure S10. LOO/LMO scaling with whiskers corresponding to the 10th and 90th percentile of the LOO/LMO value at the given training/scaled (true) training size. One perceives that values belonging to one training size have approximately the same whisker-whisker distance. For both models MLR (left) and neural network (right) the distance of the median and the 10th percentile whisker is larger than the distance of the median and the 90th percentile for smaller training sizes and these distances go to equality with growing training size. Therefore, we concluded, that the computationally feasible cross-validation (LOO or LMO) might be used for the different models, respectively.

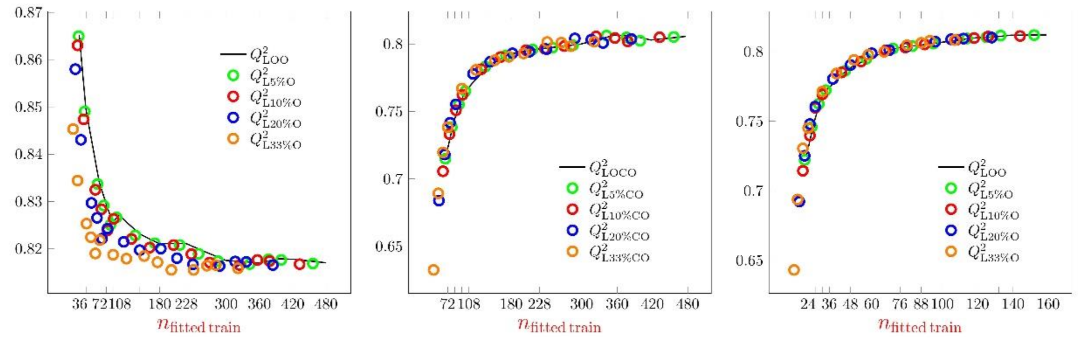

Figure S11 LOO/LMO scaling for repeated measures. The DEG dataset and its two repeats (DEG + noise) were joined into one dataset. Now each original datapoint has two repeats in the set. Assessing robustness with its validation parameters for this set (left) one obtains $Q^2_{\mathrm{LOO/LMO}}$ curves which closely resemble to the R^2 ones of the original dataset (compare result with R^2 curve for DEG in Figure 1) since two repeats or original/repeated are still present and contributing to the *RSS* and *TSS*. If one assesses robustness with leave case out validation parameters (leave out original and its two repeats, Q^2_{LOCO}, $Q^2_{\mathrm{LX\%CO}}$) then the scaling rule will be valid again (middle). As a reference on right figure the original LOO/LMO scaling rule is depicted for the DEG dataset.

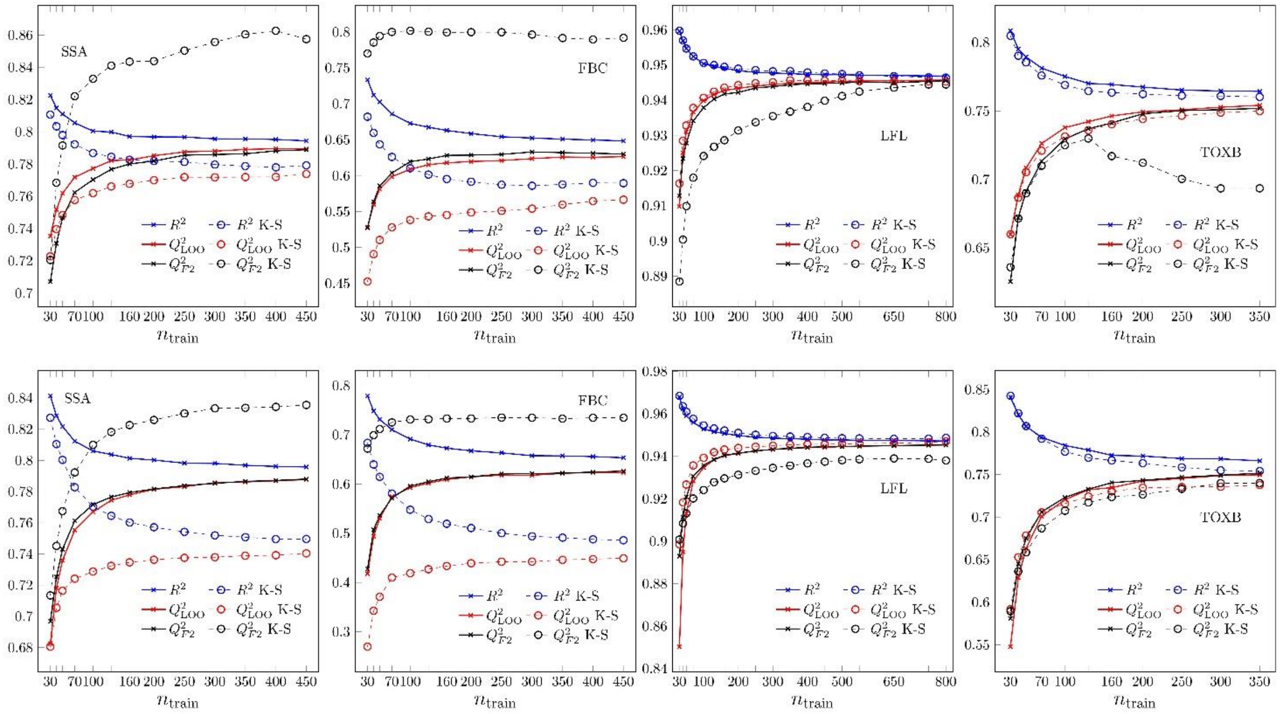

Figure S12. The figure shows the training size dependence of the Kennard-Stone (K-S) and the random train-test splitting for the split ratio of 0.8 (upper row) and 0.5 (lower row) concerning MLR modelled datasets. In all cases the Kennard Stone splitted R^2 curve is lower than the random splitted one for all training sizes and both split ratios (except for the LFL dataset where the curves trend closely). The same statement is true for the Q^2_{LOO} curves except the LFL dataset. The Q^2_{F2} curves showed a different behaviour for 50% of the investigated datasets where the Kennard Stone splitted curve was higher (~0.1, SSA) or much more higher (~0.2, FBC) than the random splitted one. Lowering the split ratio seems to lower the difference between the K-S splitted and random splitted predictivity curves and enlarges the difference in case of the goodness-of-fit and robustness curves for the datasets where K-S splitting results higher predictivity than random splitting. We concluded that Kennard-Stone algorithm changes the nominal value of the validation parameters in an unpredictable way with strong dependence on the dataset.

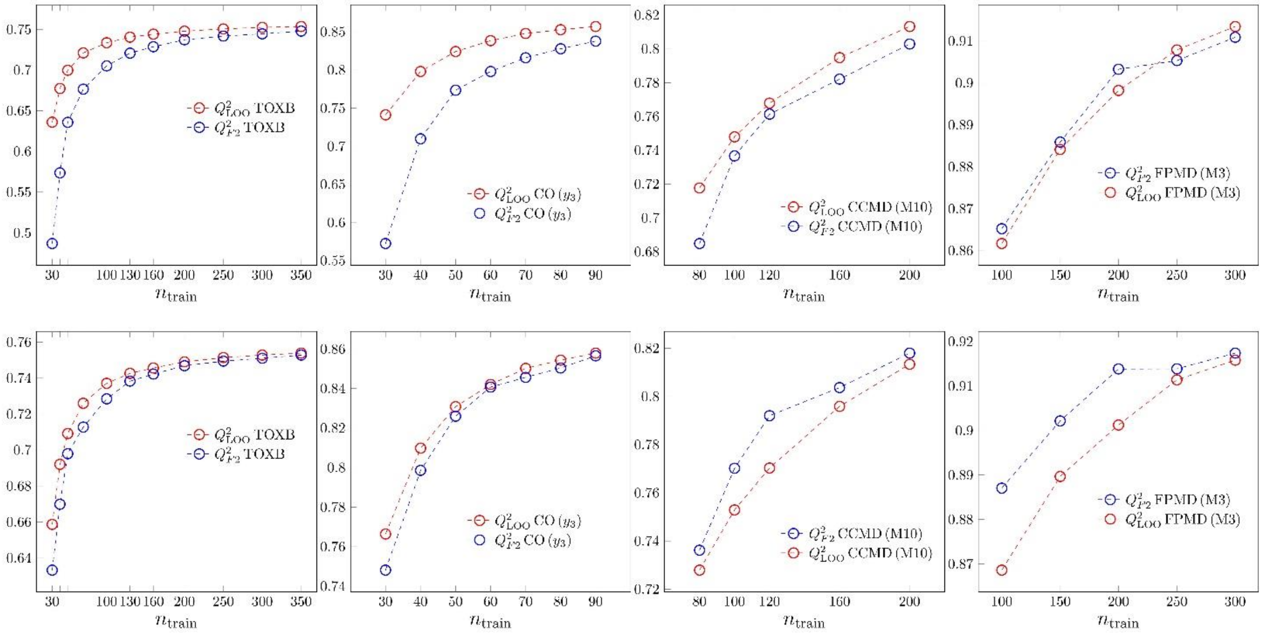

Figure S13.Distribution of Q^2_{LOO} and Q^2_{F2}. General trends are valid for the investigated datasets. For MLR and PLS2 (left and middle left) the mean (up) and the median (bottom) of Q^2_{LOO} is always larger than for Q^2_{F2} for all training sizes. In case of ANN (middle right) this trend changes for most models, the median of Q^2_{F2} is larger than the median of Q^2_{LOO}. Models without change in magnitude also occur and also models exist where mean and median of Q^2_{F2} is larger than for Q^2_{LOO}. For SVR we did not notice a general trend, for some datasets the mean curves of Q^2_{LOO} and Q^2_{F2} were very close or crossing for others the median curves. In some cases both mean and median curves of Q^2_{LOO} and Q^2_{F2} were very close.

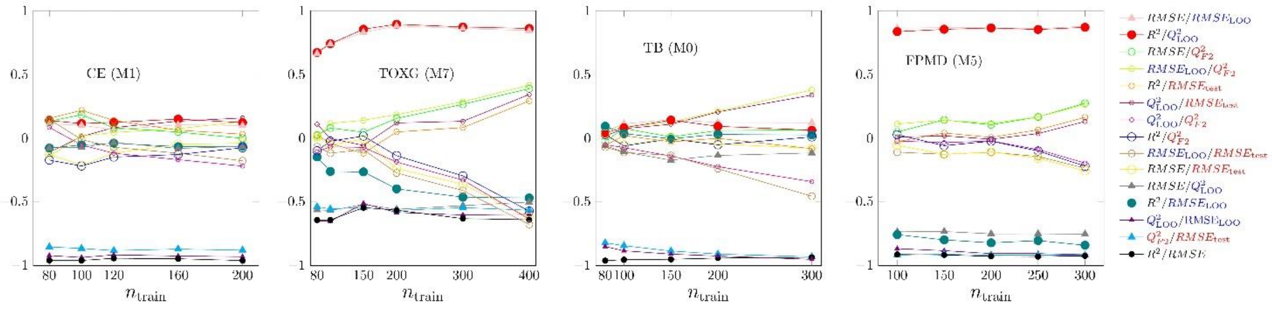

Figure S14. Sample size dependence on intra-class rank correlations. Deviations from regular cases for ANN (left ones) and SVR (right ones). For both ANN (CE (M1)) and SVR (TB (M0)) we found models that do not show the general trends observed for most models e.g. goodness of fit/robustness pairs are trending about zero for all training sizes. In some cases, models were also present which intra-class rank correlations resembled to those of MLR and PLS2 (ANN - TOXG (M7) and SVR - FPMD (M5) cf. Fig 7.).

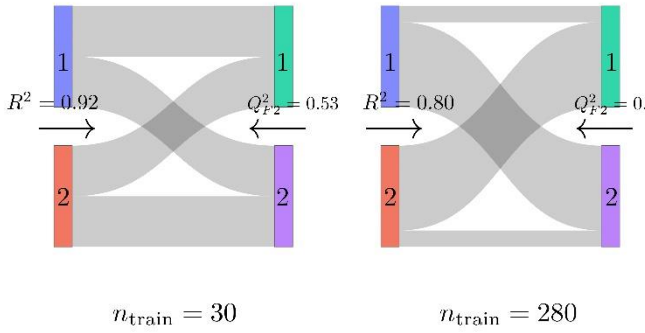

Figure S15 The R^2 and Q^2_{F2} validation parameters of the AIR y_2 response variable collected in 5000 samples were split at their median values into two groups each for training sizes 30 and 280. 4 possible group combinations are possible 1-1, 1-2, 2-1, 2-2 for each sample. For the lower training size each group (upper or lower half of the internal or external validation) contained 50% of the opposite samples. As we use up more and more data objects (close to the maximal sample size) most regular objects are placed in the training set for the better half of R^2 values and subsequently the non-regular cases are frequently present in the test set or vice versa and the group populations shrink or grow complementary. This explains also the behaviour, e.g., in Fig 7., of the R^2/Q^2_{F2} inter rank correlation which trends from 0 to -1 as with growing training size.
